# SMS Scam Detection Application Based on Optical Character Recognition for Image Data Using Unsupervised and Deep Semi-Supervised Learning

**DOI:** 10.3390/s24186084

**Published:** 2024-09-20

**Authors:** Anjali Shinde, Essa Q. Shahra, Shadi Basurra, Faisal Saeed, Abdulrahman A. AlSewari, Waheb A. Jabbar

**Affiliations:** Faculty of Computing, Engineering and Built Environment, Birmingham City University, Birmingham B4 7RQ, UK; anjali.shinde@mail.bcu.ac.uk (A.S.); shadi.basurra@bcu.ac.uk (S.B.); faisal.saeed@bcu.ac.uk (F.S.); rahman.alsewari@bcu.ac.uk (A.A.A.)

**Keywords:** unsupervised machine learning, deep learning semi supervised, feature ex-traction, smishing message

## Abstract

The growing problem of unsolicited text messages (smishing) and data irregularities necessitates stronger spam detection solutions. This paper explores the development of a sophisticated model designed to identify smishing messages by understanding the complex relationships among words, images, and context-specific factors, areas that remain underexplored in existing research. To address this, we merge a UCI spam dataset of regular text messages with real-world spam data, leveraging OCR technology for comprehensive analysis. The study employs a combination of traditional machine learning models, including K-means, Non-Negative Matrix Factorization, and Gaussian Mixture Models, along with feature extraction techniques such as TF-IDF and PCA. Additionally, deep learning models like RNN-Flatten, LSTM, and Bi-LSTM are utilized. The selection of these models is driven by their complementary strengths in capturing both the linear and non-linear relationships inherent in smishing messages. Machine learning models are chosen for their efficiency in handling structured text data, while deep learning models are selected for their superior ability to capture sequential dependencies and contextual nuances. The performance of these models is rigorously evaluated using metrics like accuracy, precision, recall, and F1 score, enabling a comparative analysis between the machine learning and deep learning approaches. Notably, the K-means feature extraction with vectorizer achieved 91.01% accuracy, and the KNN-Flatten model reached 94.13% accuracy, emerging as the top performer. The rationale behind highlighting these models is their potential to significantly improve smishing detection rates. For instance, the high accuracy of the KNN-Flatten model suggests its applicability in real-time spam detection systems, but its computational complexity might limit scalability in large-scale deployments. Similarly, while K-means with vectorizer excels in accuracy, it may struggle with the dynamic and evolving nature of smishing attacks, necessitating continual retraining.

## 1. Introduction

Smishing, a portmanteau of SMS and phishing, represents a rapidly escalating mobile security threat [1]. where attackers use text messages to deceive users by including email IDs, website links, or phone numbers to extract sensitive information or lure victims with fraudulent offers [2]. Unlike traditional email phishing, smishing leverages the ubiquity and immediacy of SMS, making it a particularly effective and dangerous attack vector [3]. The low cost of bulk SMS packages further incentivizes attackers, enabling them to launch large-scale smishing campaigns with minimal investment [4]. The urgency of addressing this threat is underscored by recent scams related to COVID-19, insurance, food deliveries, and government programs, which have resulted in significant financial losses, as reported by UK Finance [5]. The detection of SMS spam primarily involves a binary classification task, where messages are labeled as either ’spam’ or ’ham’ [6]. However, the rapidly evolving tactics employed by smishers complicate this task, necessitating continuous updates to detection methods [7]. While various techniques have been proposed for classifying SMS messages, including supervised learning models [8], these approaches often struggle to keep pace with the dynamic nature of smishing [9]. The need for constant retraining and the reliance on labeled data are significant drawbacks, particularly in real-world scenarios where new and evolving threats emerge regularly.

Existing literature on SMS spam detection has predominantly focused on supervised learning approaches, where labeled datasets are used to train models to differentiate between spam and legitimate messages [10]. Techniques such as Support Vector Machines (SVM), Naive Bayes [11], and Random Forests [12] have been extensively explored and have shown promise in detecting smishing attacks. For instance, Abayomi-Alli et al. [13] provided a comprehensive review of machine learning models used for SMS spam detection, highlighting their strengths and limitations. However, these models often require large, labeled datasets, which are not always available, and they may struggle to adapt to new types of smishing messages that differ from those seen during training. Recent research has begun to explore unsupervised and semi-supervised learning techniques as alternatives to traditional supervised methods [14]. These approaches do not rely on labeled data, making them well-suited for detecting new and previously unseen smishing attacks [15,16]. Unsupervised learning, in particular, offers the advantage of identifying anomalies in data without the need for extensive labeling, which is both time-consuming and costly [17]. Clustering algorithms, such as K-means and Gaussian Mixture Models [18], have shown potential in this area by grouping similar messages together and flagging outliers as potential spam. Rokach and Maimon [19] discussed the potential of clustering techniques in detecting unknown patterns in data, which is crucial for adapting to the ever-changing landscape of smishing.

Semi-supervised learning, which leverages a small amount of labeled data alongside a larger pool of unlabeled data, has also gained traction in recent years [20]. This approach strikes a balance between the robustness of supervised learning and the flexibility of unsupervised methods, making it particularly effective in scenarios where labeled data is scarce. Mansoor et al. [21] emphasized the need for semi-supervised methods in spam detection, noting their ability to improve model performance while reducing the dependency on labeled data. Despite these advancements, there remains a significant gap in the literature regarding the application of unsupervised and semi-supervised learning techniques specifically for smishing detection. While some studies have explored these methods in the context of general spam detection, few have focused on the unique challenges posed by smishing, such as the integration of contextual information and the detection of highly targeted attacks.

This paper aims to address these gaps by developing an AI model that leverages unsupervised and deep semi-supervised learning to detect and classify SMS messages into ’ham’ and ’spam’ categories. The rationale for choosing these methods lies in their ability to adapt to the evolving nature of smishing attacks without the need for constant retraining or large labeled datasets. Unsupervised learning techniques, such as clustering, allow the model to identify novel smishing patterns, while semi-supervised approaches enable the incorporation of limited labeled data to refine the model’s accuracy.

The proposed approach not only aligns with but also advances the current state of research by focusing on the practical application of these methods in real-world scenarios. By addressing the limitations of traditional supervised models, our work offers a more adaptable and scalable solution to the problem of smishing detection.. The structure of this paper is as follows: Section 2 presents the most recent related work, Section 3 explains the feature extraction, Section 4 explains the methodology applied, Section 5 elaborates on the results from all AI models, Section 6 shows the real-time detection and finally, Section 7 concludes the work and outlines the new directions for future research.

## 2. Related Works

The advent of smartphones has transformed communication, and the detection of SMS spam has emerged as a critical area of research. Researchers have turned to machine learning and data mining to develop effective spam filtering methods, essential for bolstering text message security and usability. Deep learning and regular expressions were employed in [22] to detect smishing messages within the UCI dataset [23]. Various preprocessing techniques, such as stemming, word stopping, and punctuation removal using regex, were applied for feature extraction. The classifier included traditional methods like multinomial NB, SVM, and RF, as well as LSTM variants for deep learning. Notably, the stacked Bi-LSTM achieved the highest accuracy, surpassing others with scores of 98.8% and 99.09%. In [24], SMS spam detection was examined using two datasets, with different classifiers and preprocessing techniques tested. The CNN classifier achieved the highest accuracy, reaching 99.19% and 98.25% accuracy for the two datasets. Authors in [25] conducted a UCI dataset study using a one-class SVM for SMS spam detection, a novel approach that outperformed traditional methods. It served as an anomaly detector, even without labeled SMS data, achieving an overall accuracy of 98%, with 100% SMS spam detection and a 3% false positive rate. In [26], a machine learning approach for SMS spam detection focused on feature extraction and evaluation, leveraging features derived from spam and legitimate message characteristics to train an averaged neural network model. This method delivered outstanding results on the UCI dataset, boasting an accuracy of 98.8% and an F-measure of 99.29%. Weka and RapidMiner were applied for spam detection on the UCI dataset in [27]. Weka SVM achieved 99.3% accuracy in just 1.54 s, while K-Means excelled in clustering with a 2.7 s runtime. RapidMiner SVM achieved 96.64% accuracy in 21 s, with K-Means taking 37.0 s for results. In [28], an intention-based SMS spam filtering method was developed, emphasizing dynamic keyword semantics. The model, which utilized 13 predefined intention labels, contextual embeddings, and supervised learning classifiers, achieved an impressive 98.07% accuracy, along with 0.97% precision and recall. The study by [29] introduced Text Augmentation for Model Improvement (TAMS) for addressing imbalanced textual data classification. TAMS employed text augmentation by replacing words with synonyms to create semantically similar messages, significantly enhancing classification accuracy. The bidirectional LSTM (Bi-LSTM) classifier achieved a high accuracy of 93.34% and an impressive F1-score of 94.18%. In [30], the UCI spam dataset was evaluated using three algorithms: back propagation neural network, naive Bayes, and decision tree. Preprocessing steps, including converting text to lowercase, removing punctuation and unique strings, stemming, and tokenization, were applied. The neural network identified the top seven smishing SMS features, achieving a final accuracy of 97.40%. The study by [31] introduced the discrete-hidden Markov model for efficient spam detection, achieving an impressive 95.9% accuracy. This model, unlike deep learning methods, is not language-specific and performs well on both Chinese and English datasets. In [32], the Gini Index metric was employed to investigate the ANN-SCG method for content-based spam SMS filtering. Experiments showcased ANN-SCG’s ability to effectively filter over a hundred spam SMS attributes swiftly, reducing memory usage. The research, which utilized datasets like DIT SMS Spam, Spam Messages Collection, and British English SMS, revealed the method’s high efficacy, achieving 99.1% accuracy in spam message filtering using just one hundred features. In [33], a combination of machine learning (NB, LR, RF, GB, SGD) and deep learning (CNN, LSTM) methods were introduced for spam filtering using UCI spam datasets. The CNN achieved a remarkable 99.44% accuracy, though the study was focused exclusively on English text messages.

Summarizing the reviewed papers, as presented in Table 1, the majority of the research focuses on supervised and deep learning algorithms, with limited exploration of unsupervised learning models. Consequently, our study aims to investigate the performance of unsupervised and deep semi-supervised models and their applicability in real-world scenarios, addressing the challenge of obtaining labeled data for training.

## 3. Feature Generation

**Figure 1:**
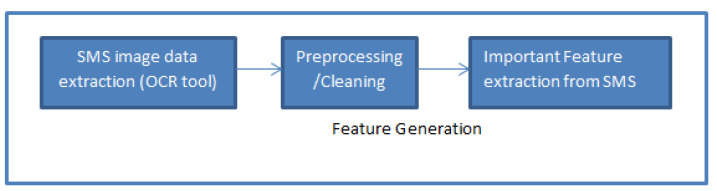
A Hierarchical framework for feature generation in the context of the proposed SMS fraud detection system.

In the realm of machine learning and artificial intelligence projects, the initial steps are pivotal for the success of model implementations [34]. The process of Optical Character Recognition (OCR) begins with capturing visual data using cameras or scanners, which act as sensing devices to detect and digitize the textual content embedded within images [35]. These sensors convert the physical properties of light and color into digital signals that represent the visual patterns of the text [36]. Once the data is captured, OCR technology processes the image, identifying and converting the detected text into machine-readable format. This foundational step is crucial, as it enables the system to transform raw image data into usable text for further analysis, such as spam detection in our proposed application. These early stages encompass data cleaning, preprocessing [37], and feature engineering [38]. Data cleaning involves the removal of extraneous elements, such as stop words, numbers, and spaces, from the raw data. Preprocessing is the phase where data is transformed and standardized to make it suitable for modeling. Finally, feature engineering entails creating essential features and evaluating their impact on model outcomes. These steps collectively lay the foundation for robust and accurate machine learning models. As illustrated in Figure 1, our research places a strong emphasis on these initial steps, recognizing them as the cornerstone in determining the ultimate success of each model implementation.

### 3.1. Dataset

**Figure 2:**
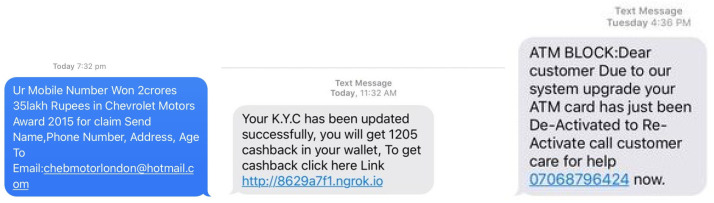
Illustrative example of a simulated SMS containing an email address, hyperlink, and contact number.

In contrast to previous studies that have primarily relied on the UCI SMS spam dataset, our research took a novel approach by collecting real user-reported spam messages captured as screenshot images using scanners. These scanned images, sourced from online platforms, were stored locally and provided a diverse and realistic dataset for our analysis. The spam messages varied in content and origin, offering a rich resource for evaluating our models. For classification, we organized the SMS spam into three distinct categories: those containing an email ID, messages with a website link, and messages that included a phone number, as visually represented in the dataset in Figure 2. To transform these image-based messages into machine-readable text, we harnessed OCR technology, leveraging the Python-Tesseract library. OCR is a sophisticated tool that detects and converts text within images into a format that computers can readily interpret. Notably, we used an open-source OCR engine maintained by Google [39]. Subsequently, we merged this new dataset of 1500 SMS messages with the UCI SMS spam dataset, which contained 5574 messages, culminating in a consolidated dataset of 7074 messages, as detailed in Table 2. This unified dataset contains a collection of English SMS text messages with varying sentence lengths, including both text and numerical content. Each record in the dataset is accompanied by a label, where ‘1’ designates ‘ham’ (non-spam) and ‘0’ indicates ‘spam’. This dataset served as the foundation for our model development and evaluation. For our unsupervised learning experiments, we fed the data without labels into the models. In the case of semi-supervised learning, we incorporated 10% of labeled data from the entire corpus of 5000 messages sourced from the UCI spam dataset, along with 90% of the newly collected SMS data from real-time users.

### 3.2. Dataset Pre-Processing

Cleaning raw data is essential for spam detection, involving the removal of stop words, numbers, spaces, and other irrelevant characters. We extract crucial features from the cleaned data to enhance classification efficiency. Data collected from various sources is often unsuitable and must be transformed into a usable format. Preprocessing starts by converting text to lowercase, facilitating natural language processing (NLP) with tools like NLTK. We utilize stopping and stemming techniques to eliminate uninformative words and then remove punctuation using Python Regex. Tokenization breaks messages into specific aspects, ready for classification models.

### 3.3. Feature Extraction

Feature extraction is crucial for spam detection, converting cleaned text into quantifiable features essential for machine learning [40]. We utilized two techniques for unsupervised and semi-supervised models in our research.

#### 3.3.1. Unsupervised: TF_IDF and PCA

TF-IDF: TF-IDF (term frequency-inverse document frequency) is a numerical metric used in statistical data analysis to assess the importance of words in a corpus. It places emphasis on both word frequency and meaning within the dataset. By reducing the significance of less important terms, TF-IDF simplifies the process of building models and helps in managing input dimensionality. The TF-IDF formula is expressed as:

(1) $$W_{i}{,}_{j}=tf_{i}{,}_{j}*log\frac{N}{df_{i}}$$

where $tf_{i}{,}_{j}$ is the number of occurrences of term *i* in document *j*, $df_{i}$ is the number of documents containing term *i*, *N* represents the total number of documents in the corpus, and the TF component counts the variety of occurrences of a term within a specific document. For example, in two instances of “Text1”, the word “part” has a TF value of 1. The “DF” stands for document frequency, indicating the number of documents in the corpus that include a specific term. For a corpus consisting of “Text1” and “Text2” with a total of two documents, the document frequency of “part” is assessed. For a comprehensive understanding of TF_IDF and its applications, please refer to Table 3 and Table 4.

PCA: PCA, a dimensionality reduction technique, simplifies datasets, while sparse PCA, a machine-learning variant, extracts main features, especially in multivariate data, by giving input features a sparse structure. It is used for reducing dimensionality while avoiding constraints. Researchers, such as [41,42,43], have employed PCA to enhance SMS spam detection by reducing feature dimensions.

#### 3.3.2. Deep Semi-Supervised: Tokenization and Sequence and Padding

Tokenization: The TensorFlow Keras Tokenizer API streamlines text vectorization by converting words into integers and creating integer or vector sequences. Punctuation is removed, reducing the number of unique words from 4520 to 3461 during preprocessing, eliminating unnecessary data for the model. Table 5 illustrates the transformation from text to tokens.

Sequencing and padding: Sequencing involves arranging sentences in order and representing them as sequences of numbers using the Tokenizer API’s texts_to_sequences() for both training and test data. To ensure consistent input shapes for deep learning models, pad_sequences() is used to make each sequence the same length. This padding can be applied either “pre” or “post,” with a specified maximum length, such as max_len=8. In experiments, max_len=25 can be applied for effective padding. Table 6 illustrates sequencing and padding and Table 7 demonstrates pre and post padding.

## 4. Proposed Work: Technical Implementation of Unsupervised and Deep Semi-Supervised Models

In our proposed work, we implemented two distinct categories of models: unsupervised learning models and deep semi-supervised learning models, each tailored for the task of smishing detection.

### 4.1. Unsupervised Learning Models

**Figure 3:**
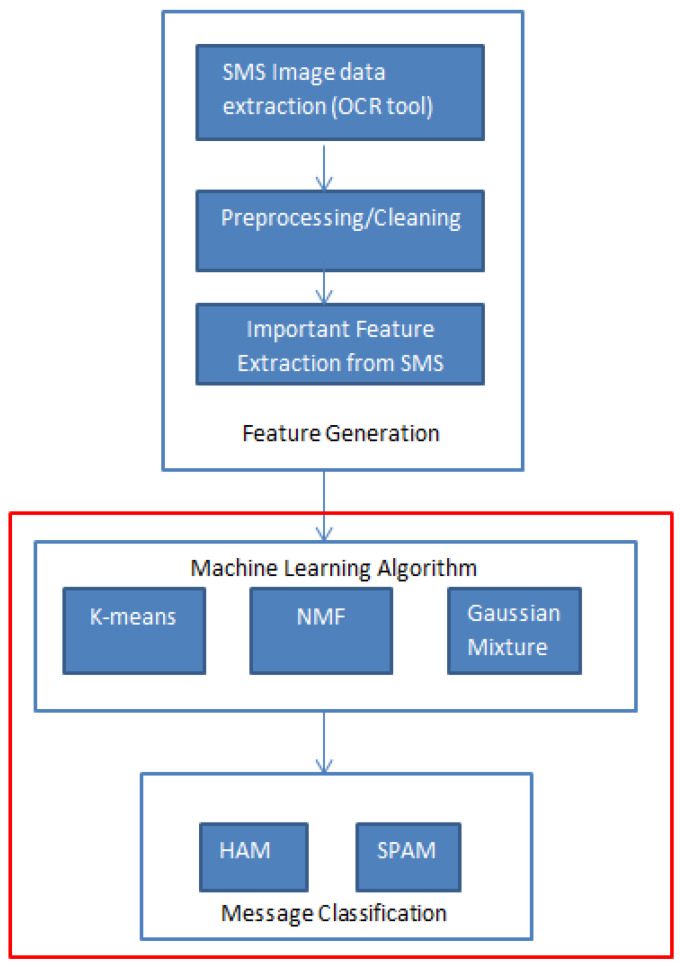
Classification framework for unsupervised methodological approaches.

Unsupervised learning is a method where models analyze and group unlabeled data, identifying patterns and distinctions without the need for human intervention. This approach is particularly useful in scenarios where labeled data is scarce or unavailable, such as in data exploration, customer insights, and image classification [44]. In our study, we employed three unsupervised models—K-Means, Non-negative Matrix Factorization (NMF), and Gaussian Mixture Models (GMM)—to classify smishing messages. The implementation is structured into three primary phases as illustrated in Figure 3:

1. Feature Generation: Features were extracted from the text messages using techniques like TF-IDF (Term Frequency-Inverse Document Frequency) and PCA (Principal Component Analysis) to capture the most relevant textual information and reduce dimensionality.
2. Machine Learning Algorithm: In this phase, the K-Means algorithm was used to partition the data into clusters based on message similarity, while NMF provided a linear combination of non-negative features, and GMM modeled the data’s distribution using multiple Gaussian distributions.
3. Message Classification: Each model classified messages as either spam or ham, based on the patterns identified during the clustering process. The performance of these models was evaluated using precision, accuracy, recall, and F1-score, providing a comprehensive assessment of their effectiveness in smishing detection.

### 4.2. Model Settings

To assess each model’s accuracy on a similar front, all experimental settings are listed in Table 8 and were kept relatively constant so that the comparisons could be made appropriately. For each model, different feature extraction was applied. We set the initial iterations as 10 and the maximum as 600. For the final comparison of all the models, random state = 99 was used to gauge accuracy and determine the best model of them all.

### 4.3. Experimental Results and Discussion

In our unsupervised approach, we processed the text data, creating distinct clusters without labels in a Python pandas dataframe. We introduced a “clusterName” column to record these clusters, enabling the extraction of classification reports, confusion matrices, and accuracy scores. During training, unsupervised classifiers processed 1500 unlabeled ham and spam messages. For testing, we used the stored model on another set of unlabeled messages and assessed its performance. In the spam classification process, we tested five different methods for unlabelled message training, as detailed in Table 8, through 20 iterations for each unsupervised ML classifier, as shown in Table 9. Interestingly, K-means Vectorizer, PCA, and Gaussian mixture exhibited varying results in each run, while NMF and K-means Transformer maintained consistent accuracy. Averaging the results, K-means Vectorizer achieved the highest accuracy at 91.01%, followed by GMM at 89.04%, and NMF at 88.24%. Meanwhile, PCA and K-means Transformer yielded similar accuracy levels, approximately 71.15% and 71.50%, respectively. After experimenting with the training data, we saved the model and feature extraction technique using the Pickle library for ease of sharing and reusability. Subsequently, we tested the trained model on unseen data to assess its performance. The K-means algorithm consistently outperformed other models, achieving the highest accuracy at 82%. Other models performed as follows on the testing data: PCA (58%), K-means Transformer (80%), NMF (80%), and GMM (81%). K-means remained the top-performing model in each experiment.

During model implementation, hyperparameter tuning was conducted to improve accuracy and message classification as shown in Table 10. Notably, min_df=10 consistently provided the highest accuracy compared to min_df=5 and min_df=14, as well as other parameters such as sublinear_tf=true, norm=l2, ngram_range=(1,2), and stop_words=‘english’. This observation held true for both the K-means and Gaussian mixture models, with the only difference being the min_df parameter set to 0. For NMF, n_component=2 and solver=mu yielded good results with 88%. The PCA model achieved 72% as its highest accuracy after dimensionality reduction with max features = 25, with higher values of 30 and 40 yielding increased accuracy but also longer runtimes. If n_component=2 was given, it returned a classification accuracy of only 38.65%; with 10, it gave 66.71%. Therefore, n_component=25 was determined as the optimal parameter for feature dimensionality reduction.

**Figure 4:**
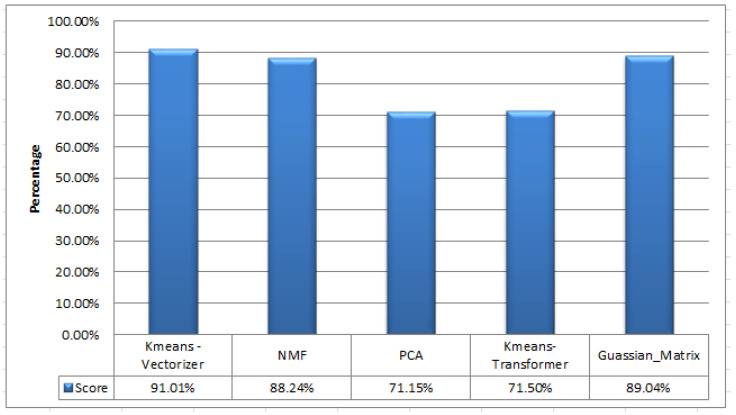
Performance metrics for unsupervised model accuracy.

Table 11 and Figure 4 reveal the performance of the different models. The K-means Vectorizer stands out with the highest accuracy at 91.01%, followed by Gaussian mixture models at 89.04%, and NMF at 88.24%. The F1-score, which strikes a balance between precision and recall, further supports the model’s effectiveness. Achieving a 90% accuracy, the model effectively detected spam SMS. However, K-means Transformer and PCA prioritized precision over recall, excelling at detecting legitimate messages, but struggling with spam identification.

### 4.4. Deep Semi-Supervised Learning

**Figure 5:**
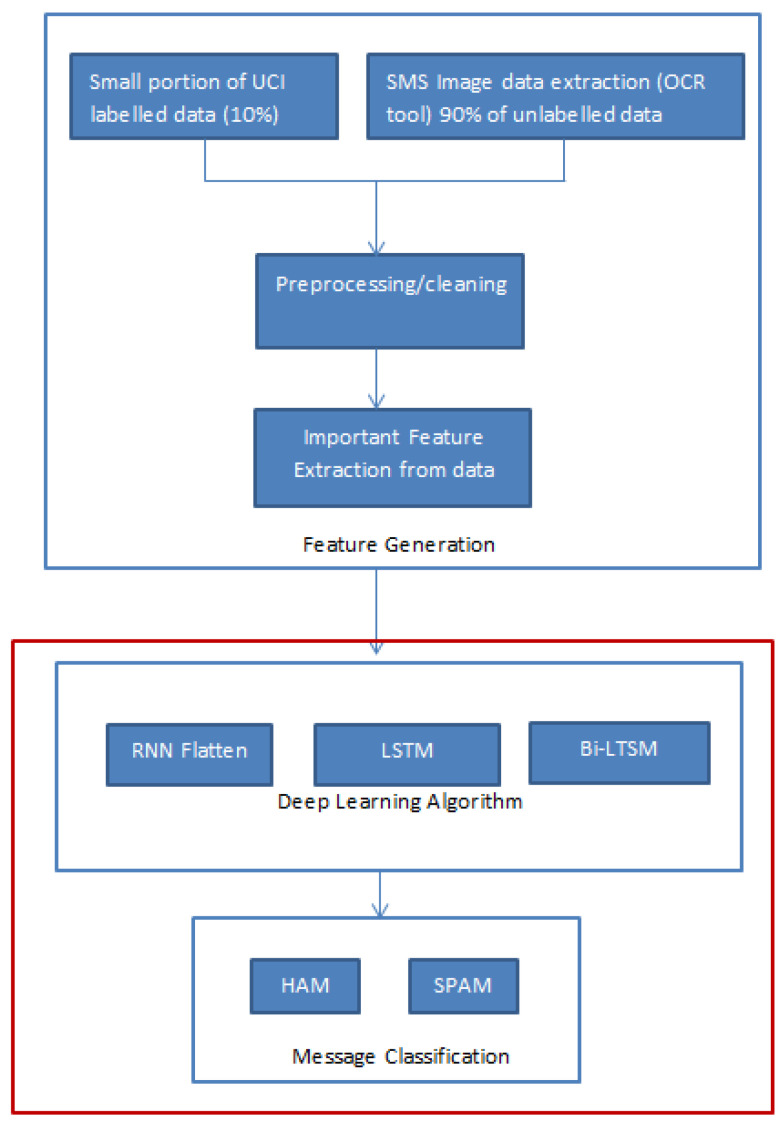
Hierarchical classification of deep semi-supervised methodological approaches.

Deep semi-supervised learning leverages both labeled and unlabeled data to train deep neural networks, which is particularly beneficial given the constraints of data annotation [45]. This approach allows the model to utilize large volumes of unlabeled data, enhancing its ability to generalize and perform well in real-world scenarios where labeled datasets are limited [46]. Our experimentation involved the deployment of three deep semi-supervised models, as outlined in Figure 5:

1. Feature Generation: Similar to the unsupervised models, this phase involved extracting features using advanced natural language processing techniques, including word embeddings like Word2Vec, GloVe, and possibly transformer-based embeddings, to capture more nuanced text representations.
2. Deep Learning Algorithm: We implemented three distinct deep learning architectures—RNN-Flatten, LSTM (Long Short-Term Memory), and Bi-LSTM (Bidirectional LSTM). The RNN-Flatten model utilized a recurrent neural network followed by a flattening layer to process the sequential data, while LSTM and Bi-LSTM models captured long-term dependencies and bidirectional context within the text.
3. Message Classification: Each of these models classified the messages as spam or ham. The classification report, which includes metrics like accuracy, precision, recall, and F1-score, was generated to evaluate the performance of these deep semi-supervised models, allowing us to compare their effectiveness in identifying smishing attacks.

### 4.5. Model Configuration Details

In Table 12, we provide details of the model architecture and its parameters. The model employs a simple linear stack architecture, specifically the sequential model. It begins with word embedding, utilizing a vocabulary of 3426 words and word vectors of length 24. The maximum sequence length is capped at eight characters. Following the embedding layer, we introduce a unit layer, and then two dense layers with 500 and 200 units, applying the ReLU activation function. These dense layers are designed to capture and process features within the data. To prevent overfitting, a dropout layer with a 50% dropout rate is included. The final layer consists of another dense layer with 100 units and 1 unit, using the ReLU and Sigmoid activation functions, respectively. This design is tailored for binary classification, distinguishing between spam and non-spam messages. During training, we employ the Adam optimizer and the binary cross-entropy loss function. The model is trained over 50 epochs with a learning rate of 0.01. Feature extraction is facilitated using Tokenizer and pad sequence techniques, which transform text data into a format suitable for neural network processing.

### 4.6. Experiments Results for Semi-Supervised Approache

We conducted experiments to assess our model’s performance in classifying spam messages using real SMS data. The algorithm was run 20 times, and the results are summarized in Table 13. Remarkably, the RNN-Flatten consistently outperformed other models in all runs. Table 14 presents a comparative performance evaluation of different classification methods. Our approach achieved an accuracy of 94.13%, with precision, recall, and F1-score all reaching 94%, surpassing other methods. Bi-LSTM achieved an accuracy of 92.78%, followed by LSTM with 92.09%. The RNN-Flatten model’s 94.13% accuracy highlights its effectiveness in spam SMS detection.

Post-experimentation, we saved the model and feature extraction technique using the Pickle library, ensuring ease of sharing and reusability. We then evaluated the model’s prediction performance on unseen data, where the RNN-Flatten algorithm led with the highest accuracy of 91%, surpassing Bi-LSTM (86%) and LSTM (84%).

**Figure 6:**
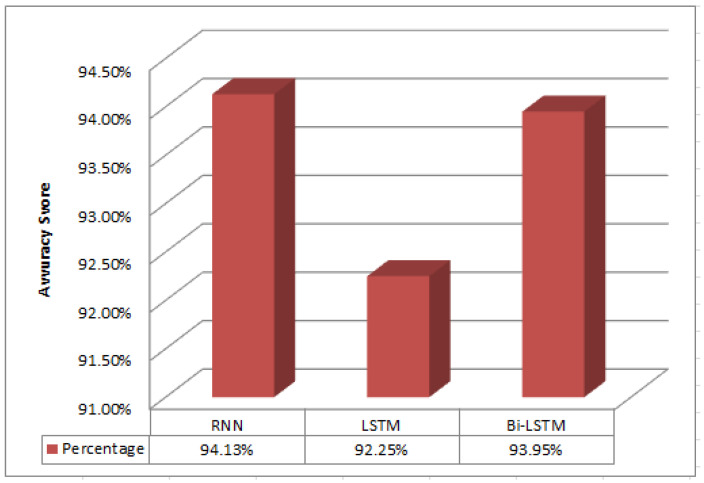
Performance evaluation of accuracy metrics for semi-Supervised models.

During model training, we found that a message_length=8 for sequence padding and word embedding produced the best results. Additionally, we observed that the RNN model with a flatten activation function consistently outperformed LSTM and Bi-LSTM across all 20 iterations, demonstrating its superiority, as depicted in Figure 6. These results highlight the efficacy of the RNN-Flatten model in spam SMS detection and showcase the importance of architecture choice in deep semi-supervised learning for text classification.

## 5. Discussion

**Figure 7:**
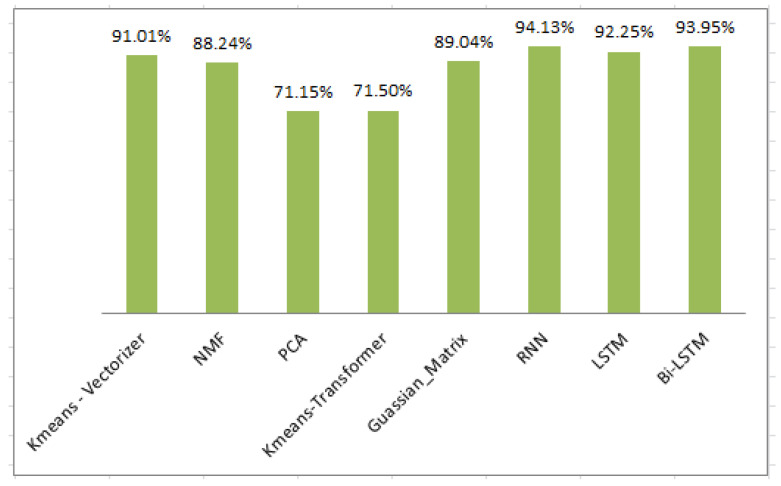
Accuracy score of unsupervised and deep semi-supervised models.

In our experiments, we evaluated multiple models for spam detection, utilizing pre-processing techniques, word embeddings, and a combination of machine learning and deep learning models. Departing from prior studies that primarily used the UCI spam dataset, we incorporated real-world, user-reported spam messages, extracted from images using Optical Character Recognition (OCR) technology. This provided a more diverse and realistic dataset for our analysis. The results indicated that the RNN-Flatten model outperformed others, achieving a notable 94.13% accuracy, compared to 91.01% with the K-means model, as shown in the accompanying Figure 7. This disparity underscores the relative strengths and limitations of each model in handling diverse and complex data.

### 5.1. Analysis of Models Performance

RNN-Flatten model: The RNN-Flatten model, a deep learning approach, benefits from its ability to capture sequential dependencies and context within the text, which is crucial for understanding the nuances of spam messages. This model is adept at learning complex patterns and relationships in data, which contributes to its superior accuracy. The deep learning architecture, with its multiple layers and non-linear activation functions, allows the RNN-Flatten model to effectively interpret and classify messages based on intricate features, including context and sequential information. Additionally, the model’s capacity to handle and learn from the sequential nature of SMS content provides it with a significant advantage over simpler clustering algorithms like K-means.

K-means Model: The K-means model, an unsupervised learning technique, relies on clustering data based on similarity measures. While it demonstrated robust performance with a 91.01% accuracy using entirely unlabelled data, it generally performs well in identifying clusters but lacks the depth of feature learning that deep learning models like RNN-Flatten offer. K-means is limited by its reliance on predefined clusters and may struggle with the complexity of nuanced text data, which affects its classification performance in comparison to models that can learn complex patterns.

### 5.2. Performance with New Data

K-means vs. RNN-Flatten on New Data: When tested with new data, the K-means model maintained an accuracy of 82%, while the RNN-Flatten model achieved a higher accuracy of 91%. This performance gap underscores the K-means model’s limitations in generalizing from training data to unseen examples. The RNN-Flatten model’s higher accuracy on new data suggests its superior capability to adapt to and generalize from diverse SMS content, owing to its deep learning architecture and feature extraction capabilities.

## 6. Real Time Detection Capabilities

**Figure 8:**
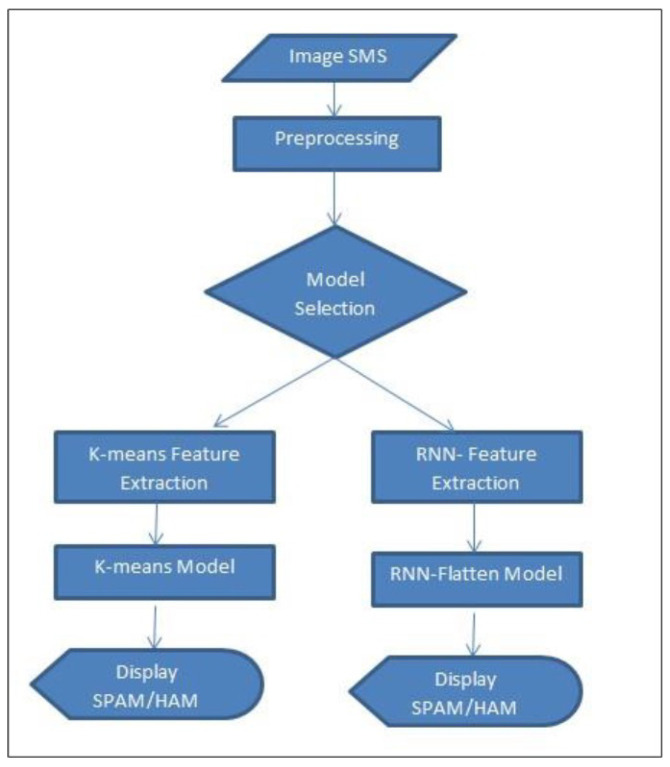
Real-Time detection and classification of SMS messages.

As illustrated in the Figure 8, the proposed framework is a streamlined web-based application designed to accept user input in real-time as an image. The application processes this image using the selected model, efficiently categorizing the message as either spam or ham. Building on the feature extraction techniques and experiments detailed in the previous sections, we developed this application using an unsupervised K-means approach and a deep semi-supervised RNN-Flatten model. This implementation allows us to evaluate the real-time effectiveness of our models in accurately detecting and classifying SMS messages. The application operates through a straightforward process designed for user flexibility and accurate detection.

**Figure 9:**
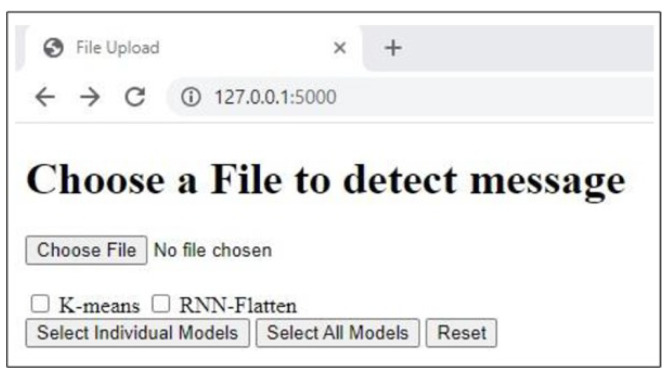
Selection of input files (image SMS).

**Figure 10:**
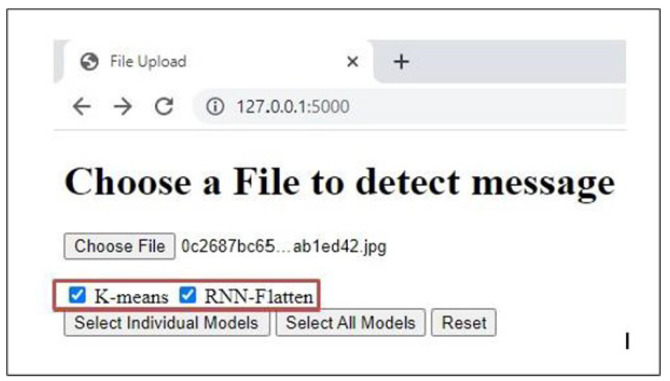
Choice of preferred model for classification.

1. First, each SMS image is individually classified, allowing the system to handle messages one at a time. The user can then select from a variety of models to analyze the nature of the image, offering the ability to choose the most suitable model for their needs as shown in Figure 9 and Figure 10 respectively.
2. Once the SMS is submitted, the system initiates preprocessing to prepare the data for analysis. Following preprocessing, the selected model’s feature extraction techniques and classifier are applied to the SMS, enabling the system to accurately assess its content.
3. Finally, the application displays the result, indicating whether the message is classified as spam or ham with the accuracy given by the selected model as shown in figure.

## 7. Conclusions and Future Work

**Figure 11:**
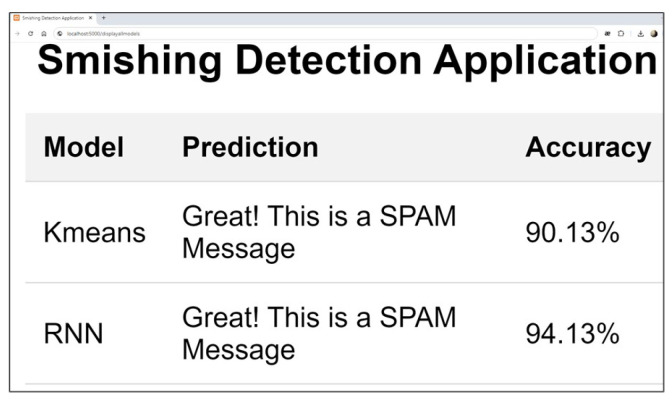
Results from both selected models.

Our research introduced a user-centric spam detection approach leveraging unsupervised and deep semi-supervised learning models as shown in Figure 11, specifically K-means and RNN-Flatten. We achieved notable accuracy rates, with K-means reaching 91.01% and RNN-Flatten achieving 94.13%. Notably, even with limited resources, our unsupervised learning model performed only 3% lower than the deep semi-supervised approach, demonstrating its efficiency and potential for practical application. We have developed a web application that enables users to detect spam from screenshots, currently supporting both K-means and RNN-Flatten models for real-time spam likelihood calculation. This application represents the first step towards integrating our models into a functional system that can be easily utilized by end-users. Moving forward, we plan to enhance the system’s capabilities by incorporating additional models and refining the user interface for better usability and visual appeal. Future developments will prioritize the following:

- Developing a more versatile application that enables users to effortlessly select and switch between different models, tailored to their specific needs.
- Enhancing spam detection accuracy by integrating advanced feature extraction methods and natural language processing (NLP) techniques, with a focus on capturing subtle details such as digits, fonts, and emojis within messages.
- Expanding language support beyond English to ensure the system’s reliability and effectiveness across a broader range of linguistic contexts.

By prioritizing these advancements, we aim to integrate our proposed model into a fully functional system that effectively meets user needs while delivering robust and adaptable spam detection capabilities, all without being hindered by the complexities of system architecture, resource allocation, or maintenance challenges.

## Figures and Tables

**Table 1:** Literature review summary.

Author	Methods	Dataset	Advantages	Challenges	Preprocessing Techniques
[22]	Multinomial NB, SVM, and RF, LSTM, Stacked LTSM, Bi-LSTM, and stacked Bi-LSTM	Stacked Bi-LSTM	98.8% and 99.09% (with and without regex)	UCI dataset	stemming, stopwords removal, regex
[24]	DT, SVM, NB, LR, AdaBoost, ANN, CNN, RF	CNN	99.19%, 98.25%	Two different Spam SMS Dataset	Tf-IDF, Tokenizer
[25]	One class SVM	One class SVM	98%	UCI dataset	TF-IDF, bag-of-words
[26]	content-based features using averaged neural networks	Neural Network Algorithm	98%	UCI dataset	C# framework for feature extraction like urls, punctuation, emoji etcs.
[27]	Classification and Clustering	For classification, SVM is best and for clustering, the K-Means algorithm is best	Weka SVM 99.3% in 1.54 s, K-Means 2.7 s, RapidMiner SVM 96.64% in 21 s, K-Means in 37.0 s.	UCI dataset	Tokenization, Stop word removal
[28]	Decision tree, SVM, Random forest	DistilBERT + SVM	98.07%	Grumble Text Website, NUS SMS Corpus (NSC), Caroline Tag’s Ph.D. Thesis, Spam SMS Corpus v.0.1 Big	BERT, DistillBERT, RoBERT, SpanBERT, NLP, Cosine Similarity Measures
[29]	RF, Bi-LSTM	Bi-LSTM	93.34%	UCI spam dataset	TAMS, Text Augumented Most similar synonymns, Word2Vec, stop words, Duplicate removal,
[30]	Back Propogation NN, NB, DT	Back Propogation NN	97.40%	UCI spam dataset	stemming, tokenization, and feature extraction for seven best features through NN.
[31]	Hidden Markov model (HMM)	HMM	95.90%	UCI spam dataset	Stop words removal, punctuation to original words
[32]	ANN, Scaled Conjugate Gradient Algorithm	ANN	99.10%	Datasets contain SMS spam, DIT spam, British language	Feature abstraction, replacement of similar words, tokenization, stemming, Lowercase conversion
[33]	NB, LR, CNN, LSTM, RF, The boosted Gradient,	CNN	99.44%	UCI spam dataset	Feature extraction

**Table 2:** Sample example of dataset.

Text	Label
Hi, How are you. When are you planning to meet me	1
Congratulations on winning the prize of 2000. To stop receiving messages, type stop www.morereplayport.co.uk, accessed on 8 August 2024 Customer Support 0987617205546	0
Good Morning. Can we discuss this issue after sometime instaed of now	1
Service announcement from BRP. You have received a BRP card. Please call 07046744435 right away to schedule delivery between 8 a.m. and 8 p.m.	0

**Table 3:** TF_IDF example.

Text1	Natural Language Processing is a part of AI
Text2	Machine learning is a part of AI

**Table 4:** TF_IDF processed example.

Vocab	AI	Is	Language	Learning	Machine	Natural	Of	Part	Process
Text1	1	1	0.317	0	0	0.317	1	1	0.317
Text2	1	1	0	0.354	0.354	0	1	1	0

**Table 5:** Tokenization example.

Before tokenizer	me also da i fel yesterday night wait til day night dear
After tokenizer	[29, 253, 319, 3, 384, 354, 200, 215, 355, 78, 200, 102]

**Table 6:** Sequencing and padding example.

Train_data	yesterday night wait	
	til day night dear	[354, 200, 215,
		355, 78, 200, 102]
Test_data	since when which side	
	any fever any vomitin	[835, 85, 349,
		3200, 120, 120]

**Table 7:** Pre and post padding example.

Encoded_train	[216, 1085, 1086, 123, 1, 1633, 320, 1634, 3, 79, 385, 2, 90, 85, 3, 40, 47]
Padded_train_pre	[0 0 0 0 0 0 0 0 216 1085 1086 123 1 1633 320 1634 3 79 385 2 90 85 3 40 47]
Padded_train_post	[216 1085 1086 123 1 1633 320 1634 3 79 385 2 90 85 3 40 47 0 0 0 0 0 0 0 0]

**Table 8:** Unsupervised learning model setting.

Model	Feature Extraction	Min_df	Clusters	Initial Iterations	Max Iterations	Random State
Kmeans	Vectorizer	10	2	10	600	99
Kmeans	Transformer	None	2	10	600	99
NMF	Vectorizer	10	2	0	600	99
PCA	Transformer	30	2	10	600	99
GMM	Vectorizer	10	2	0	600	99

**Table 9:** Outcome of the proposed models after 20 runs.

Runs	K-means Vectorizer	NMF	PCA	K-means Transformer	Guassian_Matrix
1	90.51%	88.24%	69.81%	71.87%	88.31%
2	91.88%	88.24%	71.94%	68.18%	91.47%
3	92.30%	88.24%	71.66%	68.18%	88.03%
4	91.06%	88.24%	66.78%	71.87%	82.39%
5	90.51%	88.24%	71.94%	71.87%	87.14%
6	91.27%	88.24%	71.73%	71.87%	86.59%
7	91.06%	88.24%	71.32%	71.87%	86.31%
8	92.43%	88.24%	71.87%	71.87%	92.37%
9	89.96%	88.24%	66.71%	71.87%	91.33%
10	92.57%	88.24%	72.01%	71.87%	87.28%
11	90.92%	88.24%	71.80%	71.87%	86.67%
12	89.13%	88.24%	72.01%	71.87%	85.35%
13	91.06%	88.24%	72.01%	71.87%	90.30%
14	90.44%	88.24%	69.81%	71.87%	91.47%
15	90.30%	88.24%	72.01%	71.87%	92.37%
16	90.92%	88.24%	72.01%	71.87%	92.50%
17	89.41%	88.24%	71.80%	71.87%	88.10%
18	91.54%	88.24%	71.94%	71.87%	89.61%
19	90.78%	88.24%	72.01%	71.87%	92.37%
20	92.23%	88.24%	71.80%	71.87%	90.92%
Accuracy	91.01%	88.24%	71.15%	71.50%	89.04%

**Table 10:** Model performance and hyperparameters.

Model	Hyperparameters	Feature Extraction Parameters	Accuracy
K-means Vectorizer	min df = 10	sublinear tf = true, norm = l2, ngram range = (1, 2), stop words = ‘english’	(51.03%)
K-means Vectorizer	min df = 10	-	(90.92%)
K-means Vectorizer	min df = 5	-	(88.23%)
K-means Vectorizer	min df = 14	-	(82.53%)
K-means Vectorizer	min df = none	-	(69.87%)
Gaussian Mixture	min df = 10	sublinear tf = true, norm = l2, ngram range = (1, 2), stop words = ‘english’	(50.75%)
Gaussian Mixture	min df = 10	-	(89.00%)
Gaussian Mixture	min df = 5	-	(81.00%)
Gaussian Mixture	min df = 14	-	(87.00%)
Gaussian Mixture	min df = none	-	
NMF	min df = 10	n_components = 2, solver = mu	(88.00%)
K-means Transformer	-	-	(71% )
PCA	n_components = 25	-	(72% )
PCA	n component = 2	-	(38.65%)
PCA	n component = 10	-	(66.71%)
PCA	n component = 30	-	-
PCA	n component = 40	-	-

**Table 11:** Classification report.

Model	Type	Accuracy	Precision	Recall	F1-Score
K-means Vectorizer	ML	90.92	91:00	91:00	91:00
K-means Transformer	ML	71:87	80.00	72:00	70:00
NMF	ML	88.24	88:00	88:00	88:00
PCA	ML	72.48	74:00	72.00	71:00
GMM	ML	89.40	89:00	89.50	89:00

**Table 12:** Deep semi-supervised learning model settings.

Parameter	RNN-Flatten	LSTM	Bi-LSTM
Training and Testing Ratio	80:20	8:20	8:20
Rando State	42	42	42
Vocabulary	3462	3462	3462
Max sequence leng	8	8	8
Embedding Size	24	24	24
Unit layers	1 layer(8,24)	1 layer(8,24)	1 layer(8,24)
Dense	500	500	500
Dropout	0.5	0.5	0.5
Feature Layer	Relu	Relu	Relu
Classifying Laye	Sigmoid	Sigmoid	Sigmoid
Optimizer	rmsprop	adam	adam
Loss Function	crossentropy	crossentropy	crossentropy
Training epoch	50	50	50
Feature Extraction	Tokenizer and pad sequences	Tokenizer and pad sequences	Tokenizer and pad sequence

**Table 13:** Outcome of the proposed model after 20 runs.

Runs	RNN	LSTM	Bi-LSTM
1	94.50%	92.44%	92.44%
2	95.53%	92.10%	92.78%
3	95.19%	92.44%	92.10%
4	94.85%	92.44%	93.81%
5	92.78%	91.75%	94.50%
6	94.50%	93.13%	94.50%
7	93.47%	91.41%	94.50%
8	92.44%	93.13%	92.44%
9	95.16%	91.75%	94.85%
10	94.81%	93.13%	94.16%
11	93.81%	93.13%	93.13%
12	93.13%	91.41%	94.16%
13	93.13%	91.75%	93.81%
14	93.81%	90.72%	95.53%
15	92.44%	92.10%	94.50%
16	94.16%	93.47%	94.85%
17	95.13%	93.47%	94.16%
18	93.47%	91.07%	94.16%
19	95.50%	91.75%	94.50%
20	94.85%	92.44%	94.16%
Accuracy	94.13%	92.25%	93.95%

**Table 14:** Classification report of deep semisupervised model.

Model	Type	Accuracy	Precision	Recall	F1-Score
RNN-Flatten	DL	94.13	94.00	94.00	94:00
LSTM	DL	92.09	93.00	92.00	92:00
Bi-LSTM	DL	92.78	93.00	92.00	92:00

## Data Availability

Available upon request.
